# Inhibition of Pterygium Fibroblast Migration and Outgrowth by Bevacizumab and Cyclosporine A Involves Down-Regulation of Matrix Metalloproteinases-3 and -13

**DOI:** 10.1371/journal.pone.0169675

**Published:** 2017-01-09

**Authors:** Yeoun-Hee Kim, Jae-Chang Jung, Sang Il Gum, Su-Bin Park, Jin Yeul Ma, Yong Il Kim, Kyoo Won Lee, Young Jeung Park

**Affiliations:** 1 Cheil Eye Research Institute, Cheil Eye Hospital, 1 Ayang-Ro, Dong-Gu, Daegu, Republic of Korea; 2 Korean Medicine (KM)-Application Center, Korea Institute of Oriental Medicine (KIOM), Cheomdan-ro Dong-gu, Daegu, Republic of Korea; 3 Department of Biology, College of Natural Sciences, Kyungpook National University, Daegu, Republic of Korea; University of Portsmouth, UNITED KINGDOM

## Abstract

We examined the connection between matrix metalloproteinase (MMP) expression/activity and pterygium fibroblast migration, and how these were affected by bevacizumab and/or cyclosporine A (CsA). Fibroblasts were obtained from 20 pterygia and 6 normal conjunctival specimens. Expression levels of MMP-3 and MMP-13 were examined after bevacizumab administration. Immunofluorescence staining was used to examine expression of both MMPs in fibroblasts migrating out from explanted pterygium tissues. Rates of cell migration from explant-cultured pterygia tissues and scratch-wounded confluent pterygium fibroblasts were examined in the presence of MMP-3 or MMP-13 inhibitors, as well as bevacizumab and/or CsA. A scratch wound healing migration assay was performed to determine the effects of bevacizumab and/or CsA. Protein expression of both MMPs in pterygium tissues and in cells migrating from organ-cultured pterygium tissues was greater than that observed in normal cells. Inhibition of the activities of both MMPs decreased their expression levels; these were also significantly reduced in bevacizumab-injected pterygium tissues. Bevacizumab significantly reduced the expression of both MMPs and cell migration. Pretreatment with CsA prior to bevacizumab exposure markedly inhibited cell migration and the expression of both MMPs. CsA enhanced the inhibitory effects of bevacizumab on pterygium fibroblast migration *in vitro*, possibly by inhibiting expression of both MMPs. These findings suggest that combined CsA and bevacizumab treatment may provide a potential therapeutic strategy for reducing the rate of pterygium recurrence.

## Introduction

Pterygium is a common ocular surface disease in humans that is attributed to chronic ultraviolet-B exposure and can cause vision loss. It results from the migration of abnormal limbal basal epithelial stem cells (pterygium cells) into Bowman's layer (BL) and causes the dissolution of this layer. Pterygium is characterized by high vascularization, proliferation, invasive ocular surface lesions, and aberrant extracellular matrix (ECM) remodeling [1–5]. Surgical excision is the standard treatment for a pterygium, but despite advances in surgical techniques, the pterygium recurrence rate is high [6–11].

Matrix metalloproteinases (MMPs) are zinc-dependent endopeptidases that are able to degrade all components of the ECM in connective tissues [12, 13]. They are important in various physiological and pathological processes, including tissue remodeling, wound healing, angiogenesis, cell invasion, and differentiation [14]. It has been suggested that MMPs play a role in pterygium formation because excessive fibroblast proliferation and invasion occur at the head of a pterygium, with BL and corneal stroma destruction [15]. Pterygium cells produce the MMPs that dissolve BL, thus stimulating the growth of stromal fibroblasts [4]. The over-expression of MMPs in altered limbal basal epithelial cells (pterygium cells) may be the primary cause of pterygium progression [16]. ECM modulation by MMPs may occur early in the course of pterygium invasion [3, 17]. Given that many studies have shown a strong association between MMPs and tumor progression/invasion [18], the invasive nature of the pterygial lesion is likely to involve altered MMP activity.

MMPs are expressed by a variety of cell types and they are categorized into five groups, based on their substrate specificity and cellular localization. These groups are the collagenases (MMP-1, -8, -13), the gelatinases (MMP-2, -9), the stromelysins (MMP-3, -10, -21, -22), the membrane-type MMPs (MT-MMPs), and the others [1, 19]. Increased levels of MMP-1, MMP-2, MMP-3, MMP-7 (matrilysin), MMP-8, and MMP-9 have been reported in pterygia [1, 5, 14, 20–25]. In particular, pterygium cells may also cause the activation of fibroblasts at the head of the pterygium, leading to cleavage of the fibrillar collagen in BL due to the production of high levels of active MMP-1 and MMP-3 [22, 24]. Expression of MMP-1 has been detected in pterygium fibroblasts found between the corneal epithelium and BL at the edge of the advancing pterygium [26]. Elevated levels of several other MMPs (MMP-2, MMP-3, MMP-7, MMP-8, MMP-9, and MMP-14) have also been detected in the pterygium, particularly at the advancing head [17, 24, 25]. The increased expression of multiple types of MMP and their release by pterygium cells facilitate invasion by degrading components of the BL and the adjacent stromal matrix [22, 26, 27]. Furthermore, it has been suggested that ECM degradation releases a range of cytokines including vascular endothelial growth factor (VEGF) and fibroblast growth factor; these stimulate angiogenesis, as well as the migration and proliferation of pterygium cells [14].

VEGF is involved in angiogenesis and has been detected in pterygia [28–31]. MMP-3 has also been clearly implicated in angiogenesis because it influences the generation of angiostatin, and an MMP-3 inhibitor was shown to inhibit angiogenic responses [32–34]. Importantly, MMP-3 can degrade many ECM because it has broad substrate specificity or activates other pro-MMPs such as MMP-1, MMP-9, and MMP-13, and it also plays a role in cell migration and angiogenesis [5, 35, 36]. During wound healing, MMP-13 is involved in a range of biological functions that include inflammatory responses, cell movement, angiogenesis, and proteolysis [37]. Recombinant MMP-13 also promotes the secretion of VEGF-A from fibroblasts and endothelial cells, and MMP-13 was suggested to directly and indirectly promote tumor invasion and angiogenesis, both *in vitro* and *in vivo* [38]. MMP-13 mediates collagenolysis-dependent angiogenesis *in vivo* [39]. Decreased tumor growth in MMP-13(-/-) mice was associated with reduced blood vessel density [40], and a lack of MMP-13 reduced the vascular density of wound granulation tissue [41]. The diminished expression of MMP-13 in hypertrophic chondrocytes inhibited growth plate angiogenesis [42]. MMP-13 has recently been implicated in corneal vascularization [43]. In addition, MMP-13 contributes to experimental choroidal neovascularization [43] and acts as a stromal mediator in controlling persistent angiogenesis in skin carcinoma [44].

Bevacizumab is a well-known angiogenesis inhibitor that slows the growth of new blood vessels. This recombinant humanized monoclonal antibody binds to all types of human VEGF, thus preventing the interaction between VEGF and its receptors on the surface of endothelial cells [45]. Bevacizumab has been used to treat choroidal neovascularization, and more recently for diabetic macular edema [46–51]. Recent studies have demonstrated that subconjunctival bevacizumab injections are useful in the management of patients with recurrent pterygium [52–58].

Cyclosporine A (CsA) is one of the most promising immunosuppressive drugs and is widely used to prevent tissue rejection following organ transplantation [59]. CsA can be administered topically or by a subconjunctival injection to treat a variety of inflammatory disorders of the ocular surface [59–61]. CsA prevents the activation and nuclear translocation of cytoplasmic transcription factors that are required for T-helper cell activation and inflammatory cytokine production [62]. Importantly, topical application of CsA prevents pterygium recurrence [63–66]. A previous study suggested that CsA treatment of the residual conjunctiva after pterygium excision may block the activation and proliferation of pterygium fibroblasts [25]. Both in primary and recurrent pterygium, CsA is an effective inhibitor of fibroblast proliferation in culture [67]. It also inhibits endothelial cell proliferation and angiogenesis [68]. However, it is well known that CsA treatment can result in a number of potentially serious adverse drug reactions (ADRs). The risk of these ADRs increases with the CsA dosage and treatment time. Therefore, for safety, a low CsA concentration (0.05%) has been used for the treatment of many ocular conditions [69–74].

Although many MMPs were expressed in pterygium tissues, our study mainly focused the activities of MMP-3 and MMP-13 which may play an important role in the process of pterygium progression. Recently, we reported that CsA down-regulated MMP-3 and MMP-13 expression in cultured pterygium fibroblasts [75]. Interestingly, bevacizumab significantly reduced the expression of MMP-1 in cultured Tenon’s fibroblasts from primary and recurrent pterygium [76]. These studies prompted us to investigate whether bevacizumab down-regulates the expression of MMP-3 and MMP-13 in pterygium tissues *in vivo*, and decreases the migration of pterygium fibroblasts *in vitro*. Furthermore, we compared the inhibitory effects of CsA and bevacizumab on the regulation of MMP expression and migration of pterygium fibroblasts *in vitro*.

## Materials and Methods

### Ethics Statement

This present study was carried out with the approval of the Human Study ethics Committee at Cheil Eye Hospital, Daegu, Korea, and the specimens were handled in accordance with the tenets of the Declaration of Helsinki. Written informed consent was obtained from all participating patients.

### Specimens from Patients

Specimens of pterygium tissue were collected from Korean patients (10 males and 10 females) during pterygium surgery. All specimens were taken from the head of the pterygium, which had invaded the central cornea. Normal conjunctival tissues, to be used as a control, were collected from the conjunctiva of 6 patients (3 males and 3 females) during cataract surgery.

### Reagents

Bevacizumab was purchased from Genentech Inc. (San Francisco, CA, USA) and CsA (0.05% Restasis^®^) was purchased from Allergan Inc. (Irvine, CA, USA). MMP-3 inhibitor VII and an MMP-13 inhibitor were purchased from Calbiochem (San Diego, CA, USA). The anti-MMP-3 antibody was purchased from Chemicon International Inc. (Temecula, CA, USA) and the anti-MMP-13 antibody was purchased from Abcam (Cambridge, MA, USA). Anti-β-actin was purchased from Santa Cruz Biotechnology Inc. (Santa Cruz, CA, USA). 4',6-Diamidino-2-phenylindole (DAPI) and Prolong Gold were purchased from Invitrogen (Carlsbad, CA, USA).

### Preparation of Human Tissues and Cells

Fibroblasts were obtained from pterygium and normal conjunctival specimens using the explant methods reported by Li et al. [24]. Immediately after excision, the tissue was placed in either Hanks’ balanced salt solution or phosphate-buffered saline (PBS) containing 1% penicillin/streptomycin (Gibco BRL, Gaithersburg, MD, USA). The tissues were finely minced into 1-mm^3^ pieces and placed in six-well culture plates with 1 ml of Dulbecco’s modified Eagle’s medium (DMEM)/F12 (1:1 vol/vol) containing 10% fetal bovine serum (FBS; Gibco BRL, Gaithersburg, MD, USA), ITS (5 μg/ml insulin, 5 ng/ml selenium, 5 μg/ml human transferrin; Sigma), and 1% penicillin/streptomycin. Seeded tissues were incubated at 37°C in a humidified air atmosphere with 5% CO_2_ and fed daily. When the cells began to form a monolayer, the tissue pieces were removed. When the conjunctival fibroblast cultures reached confluence, they were detached from the dishes using trypsin-EDTA and re-plated into new dishes at a ratio of 1:3. Primary human pterygium and conjunctival fibroblast cells (passages 4 and 5) were used in these experiments.

### Paraffin Embedding and Immunohistochemistry

Pterygium and normal conjunctiva tissues were harvested in cold PBS, fixed in 4% paraformaldehyde (PFA) in PBS for 24 h at 4°C, and then washed twice in PBS. The samples were then dehydrated through an ethanol series, cleared by soaking in xylene, embedded in paraffin, and sectioned (5 μm) using a microtome RM 2125RT (Leica, Wetzlar, Germany). Slides containing paraffin sections were deparaffinized in xylene and rehydrated through an ethanol series, and endogenous peroxidase activity was inactivated by incubation in 0.3% H_2_O_2_ in methanol for 10 min. The sections were then rinsed in 0.1 M tris-buffered saline (TBS; pH 7.4) and boiled in citrate buffer (pH 6.0) containing 0.3% Tween-20 for 4 min. Finally, the sections were incubated with a blocking solution (5% normal goat serum and bovine serum albumin in TBS) at room temperature (RT) for 1 h, and subjected to indirect immunohistochemistry using an antibody against MMP-3 or MMP-13 for 1 h. For the negative control, the primary antibody was omitted and the slides were incubated with the blocking solution. Next, sections were incubated with the corresponding secondary antibodies conjugated with biotinylated anti-rabbit IgG and with Vectastain ABS reagent (Vector Laboratories, CA, USA) for 1 h. Then, sections were incubated with a substrate from the VECTOR1 NovaRED substrate kit (Vector, Burlingame, CA), and counterstained with hematoxylin. The sections were dehydrated, cleared, and mounted with Permount (Fisher, Fair Lawn, NJ). Images were captured using a Zeiss microscope (Axio Vision 4; Carl Zeiss, Jena, Germany). Quantification of the immunohistochemical signal was performed by calculating the percentage of the stained area using ImageJ software (http://openwetware.org/wiki/Sean_Lauber:ImageJ_-_Threshold_Analysis).

### Immunofluorescent Staining of Cells Migrating from Tissue Explants

When the cells began to form a monolayer, the tissue pieces were removed and fixed with 4% PFA in PBS (pH 7.4) at RT for 15 min. The fixed cells were permeabilized with 0.3% Triton-X for 10 min, and blocked overnight with 5% normal goat serum and BSA in TBS at 4°C. The cells were then incubated overnight with monoclonal antibodies against MMP-3 or MMP-13 (diluted 1:100) in 5% BSA, and washed three times in TBS. The cells were further incubated with the appropriate Alexa 488-conjugated secondary antibodies at RT for 1 h, and nuclei were counterstained with DAPI (1 μg/ml). The cells were washed three times with PBS and mounted prior to image capture using a Zeiss fluorescence microscope (Axio Vision 4; Carl Zeiss, Jena, Germany). The green fluorescence density was measured in a color histogram using the ImageJ software program.

### Cell Migration and *In Vitro* Scratch Wound Assay

Passage 4 pterygium fibroblasts (3 × 10^5^ cells/well) were seeded on 6-well plates in 2 ml DMEM/F12 (1:1 vol/vol) containing 10% FBS and allowed to adhere for 24 h. Cells were then washed and incubated with serum-free medium. At this time, a scratch was made through the center region of the confluent sheet using a yellow pipette tip; suspended and detached cells were then washed out with the serum-free medium. Indentations were made within the wound area to establish points of reference [77]. The cells were then exposed to MMP-3 inhibitor VII (1 μM), an MMP-13 inhibitor (1 μM), or bevacizumab (1 μg/ml) in serum-free DMEM-F12 medium, and maintained for 24 or 48 h without a medium change. Cells were exposed to CsA (1 or 100 μg/ml) for 3 or 10 min prior to incubation with fresh serum-free medium, with or without bevacizumab, for 24 or 48 h. Images were captured at the same position within the wound region and the cell migration rate was determined using ImageJ.

### Measurement of Cell Migration from Tissue Explants

When cells began to migrate, they were exposed to MMP-3 inhibitor VII, an MMP-13 inhibitor, bevacizumab, and/or CsA. Cell migration was captured daily by phase contrast microscope for three days.

### Subconjunctival Administration of Bevacizumab

This procedure was conducted at the Cheil Eye Hospital in Daegu, on patients with primary pterygium. One surgeon (Y.J. Park) performed all of the surgeries using previously described surgical techniques [78, 79]. The eyes were anesthetized with topical proparacaine hydrochloride drops (Alcaine, Alcon) and visualized under a microscope in order to administer a subconjunctival injection of 2.5 mg/0.1 ml bevacizumab on the pterygium body using a 1-ml syringe with a 30-gauge needle [78, 79]. After the subconjunctival injection of bevacizumab, the patients were treated with topical levofloxacin (Cravit, Santen Pharmaceutical, Osaka, Japan) eye drops 4 times/day for 1 week. Then, the pterygium surgical technique reported by Kim at al. [80] was performed on each patient. After the tissue was harvested, it was fixed with 4% PFA.

### Western Blot Analysis

For lysate preparation, tissues or cultured cells were washed twice with PBS and then placed in ice-cold radioimmunoprecipitation assay (RIPA) buffer containing a protease inhibitor cocktail (Sigma, St. Louis, MO). The lysate protein concentration was determined using a bicinchoninic acid assay kit. Western blot analysis was performed using standard techniques. Equal amounts of lysate protein (30 μg) in the RIPA buffer were separated by 8% sodium dodecyl sulfate-polyacrylamide gel electrophoresis under reducing conditions, and the proteins were electrophoretically transferred to nitrocellulose membranes. The membranes were blocked in 5% non-fat dried milk in TBS containing 0.1% Tween-20 (TBS-T) for 1 h, and then incubated overnight at 4°C with primary polyclonal antibodies raised against MMP-3, MMP-13, or β-actin; these were all diluted in TBS-T containing 5% dried milk. The primary antibodies were detected by incubation with horseradish peroxidase-conjugated secondary antibodies for a further 1 h. Specific antibody binding was visualized using an enhanced chemiluminescence detection kit (ELPIS Biotech, Korea) and X-ray film exposure. The presented data were representative of at least three independent experiments. Densitometric analyses were conducted using Image J software.

### Statistical Analysis

Each experiment was repeated three or more times. Data were evaluated by one-way analysis of variance (ANOVA) and Turkey’s test or by two-way ANOVA. The analyses were performed using GraphPad PRISM software^®^ (GraphPad PRISM software Inc., Version 5.02, CA, USA). Results are expressed as the mean± the standard error of the mean, and P values of < 0.05 were considered statistically significant.

## Results

### Immunohistochemical Localization of MMP-3 and MMP-13 in Human Conjunctival and Pterygium Tissues

**Fig 1:**
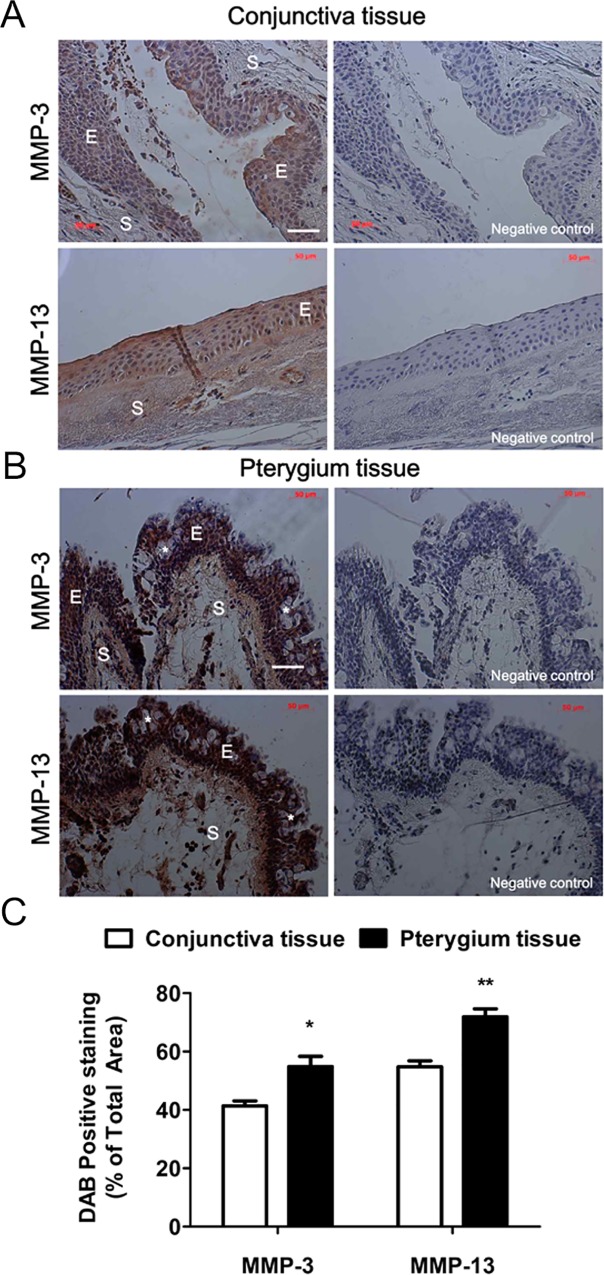
Expression patterns of MMP-3 and MMP-13 proteins in human conjunctival and pterygium tissues. Representative immunohistochemical images of MMP-3 and MMP-13 protein signals in paraffin-embedded sections of normal conjunctiva (A) and pterygium (B) tissues. Negative controls that were incubated with secondary antibodies, but not with primary antibodies, showed no staining (right panels). All tissue sections were also counterstained with hematoxylin. There were numerous surface epithelial goblet cells (*). E: epithelia, S: stroma. White scale bars: 50 μm. (C) Quantification of the immunohistochemical signal, expressed as the percentage of the total area that was positively stained. Data shown are the mean ± standard deviation, with 3 mice in each group. *P < 0.05, **P < 0.01 vs conjunctiva tissue.

To examine the association of pterygium with MMP-3 and MMP-13 protein expression, immunohistochemistry was performed on normal conjunctival and pterygium tissues (Fig 1). In the normal conjunctiva, the epithelium regularly consisted of 5-6 cell layers (Fig 1A). In contrast, irregular and hyperplasic multilayers of epithelium were seen in the pterygium groups (Fig 1B). We identified numerous surface epithelium goblet cells (*), grouped as intraepithelial glands with foamy cytoplasm (Fig 1B). In the pterygium tissue, MMP-3 and MMP-13 expression levels were significantly stronger than those observed in normal conjunctival tissues. The stained areas were 41.37-54.77% (MMP-3) and 54.81-71.90% (MMP-13) larger in the pterygium tissue than in the normal conjunctival tissue (Fig 1C). Interestingly, expression of both MMP-3 and MMP-13 proteins was detected in the epithelium and stroma of the pterygium tissue, but these were weakly expressed in normal conjunctival tissue (Fig 1A). Notably, as compared to the MMP-3 staining pattern, more intense MMP-13 staining was detected in the superficial epithelial layer of the pterygium tissue.

### Correlation Between MMP-3 and MMP-13 Expression and Pterygium Fibroblast Migration

**Fig 2:**
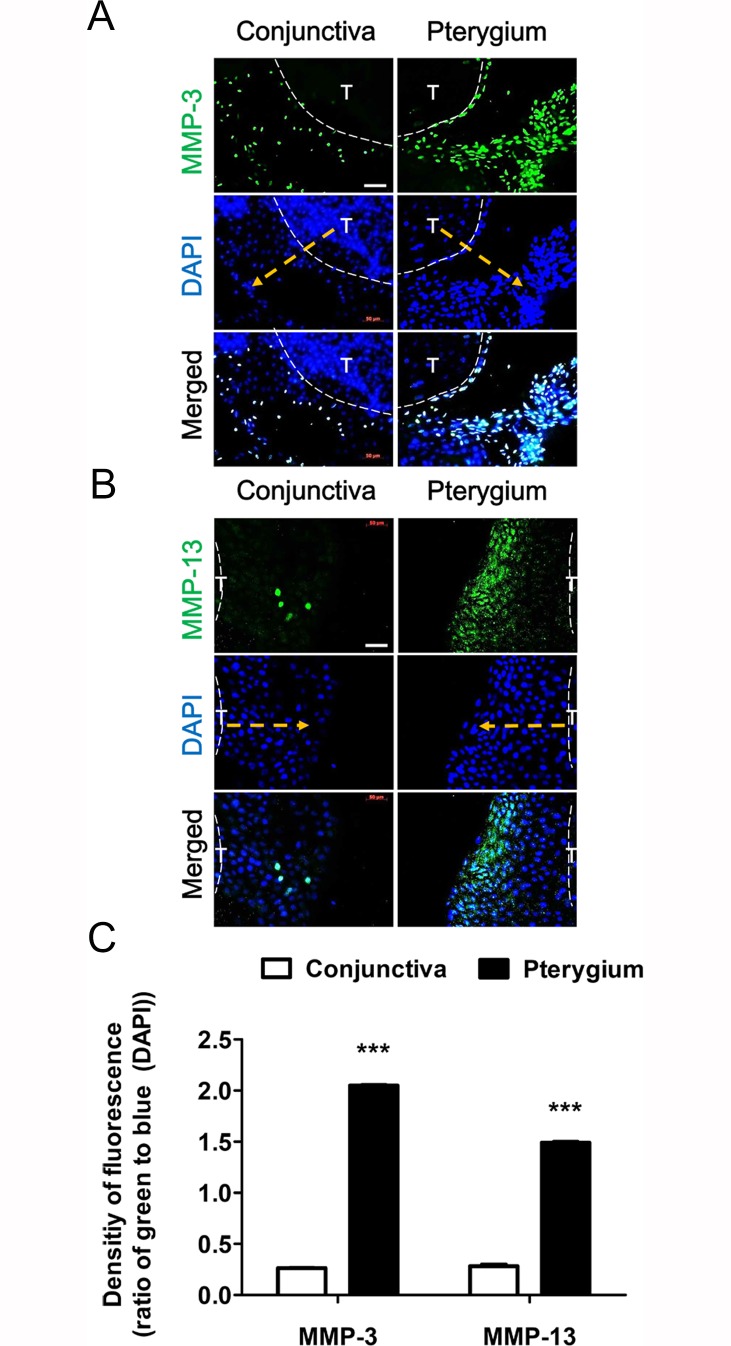
Correlation between MMP expression and fibroblast migration from conjunctival and pterygium tissue explants. (A) MMP-3 and (B) MMP-13 proteins are shown as green fluorescence (day 3 in culture), and nuclei are counterstained with DAPI (blue). After removal of the explant, cells were fixed and stained with the appropriate antibody. The direction of the arrow (dotted yellow) indicates cells migrating out from the explant (inside the dotted white lines). T: Tissue. White scale bars: 50 μm. (C) The density of green-fluorescence to blue was represented by ImageJ using a color histogram. Data represent the mean ± standard deviation of 3 separate experiments. ***P < 0.001 vs control.

Based on the immunohistochemical data (Fig 1), we examined whether fibroblasts migrating from the pterygium explant culture expressed high levels of MMP-3 and MMP-13. Fig 2 shows that normal conjunctiva fibroblasts had started to migrate out from the cultured specimens by day 3. Both conjunctiva and pterygium specimens were removed just before staining (dotted white line). Immunofluorescence staining of conjunctiva tissue identified few migratory cells that expressed MMP-3 (Fig 2A) and MMP-13 (Fig 2B). On the other hand, many more migratory cells from the pterygium specimen expressed MMP-3 and MMP-13 (direction to dotted yellow arrow) (Fig 2). In addition, DAPI staining confirmed that many migratory cells had grown out from the edge of the pterygium tissue. As expected, fewer cells remained in the region that the pterygium tissue had been removed from inside the dotted white lines. In contrast, many cells were not migratory and thus remained in the region that the conjunctiva tissue had been removed from just before staining (Fig 2). To quantify the number of migrating fibroblasts expressing both MMP-3 and MMP-13 proteins, we created a bar graph to summarize the relevant data. The resultant pixel value was 1.5-2-fold higher for the pterygium tissue explant than for the normal conjunctiva tissue (Fig 2C).

### Effects of MMP-3 or MMP-13 Inhibition on Pterygium Fibroblast Migration

**Fig 3:**
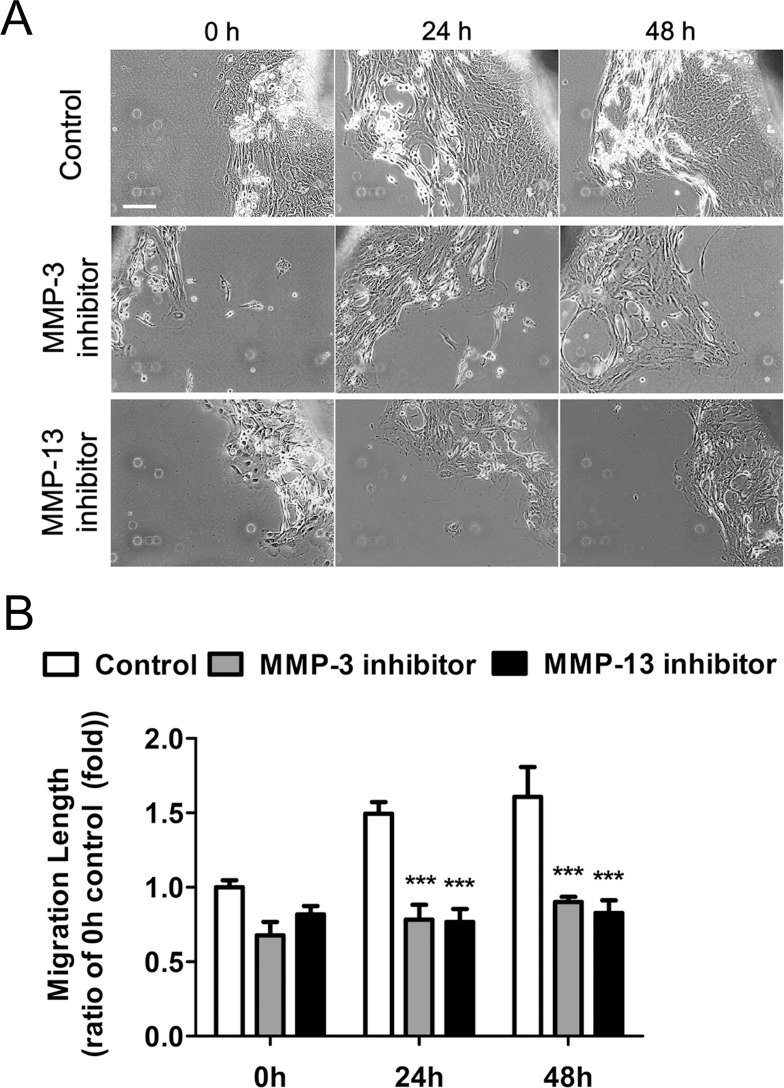
Inhibition of MMP-3 or MMP-13 activity reduced cell migration from pterygium explants. The effect of MMP inhibition on cell migration was determined by measuring the length of migration from the explanted tissue. When cells began to migrate from the explant, they were exposed to MMP-3 inhibitor VII and an MMP-13 inhibitor. (A) Cell migration was captured using a phase contrast microscope after treatment with the indicated inhibitor for 0 h, 24 h, and 48 h. White scale bar: 50 μm. Cell outgrowth from the explanted tissue in the control group increased continuously during the culture period. In contrast, the rate of cell migration was greatly reduced by treatment with an MMP-3 or MMP-13 inhibitor. (B) Migration rates under the indicated conditions. Migration was quantified under 0 h control condition. ***P < 0.001 vs the corresponding control value.

The fibroblasts migrating from pterygium tissue explants produced both MMP-3 and MMP-13 proteins (Fig 2), and we therefore examined whether inhibition of either of these activities affected fibroblast outgrowth from the explanted pterygium for 2 days (0 h) (Fig 3). Thereafter, pterygium explants were cultured in serum-free medium in the presence of MMP inhibitors for 24 and 48 h. As the culture time increased, the number of fibroblasts that grew out from the tissue edge gradually increased under non-treated control culture conditions (Fig 3A). On the other hand, treatment with either an MMP-3 or MMP-13 inhibitor greatly inhibited fibroblast outgrowth from the tissue edge over time. Quantification of fibroblast outgrowth from the tissue edge also found a significant reduction in the presence of an MMP-3 or MMP-13 inhibitor (Fig 3B). Taken together, these data indicate that fibroblast outgrowth from the pterygium tissue edge was mediated, at least in part, by MMP-3 and MMP-13.

### Effects of MMP-3 and MMP-13 Inhibition on Pterygium Fibroblast Migration into a Scratch Wound

**Fig 4:**
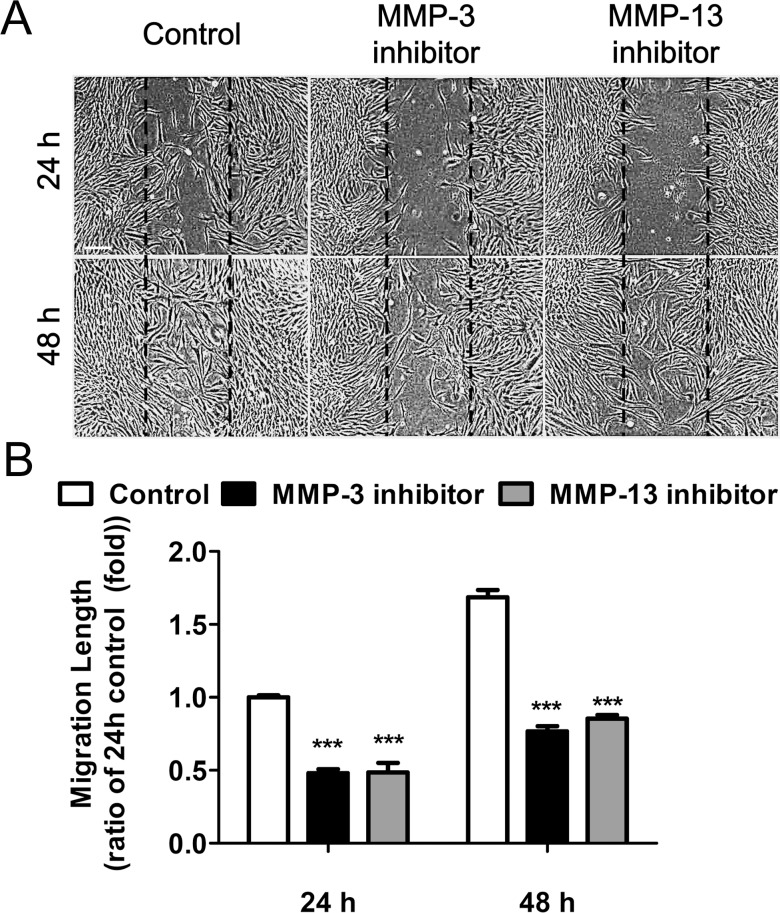
Inhibition of MMP-3 or MMP-13 activity reduced pterygium-derived fibroblast migration. Pterygium fibroblasts were treated with an MMP-3 (1 μM) or MMP-13 (1 μM) inhibitor in serum-free medium, directly after the scratch wound. (A) Representative phase contrast microscopy images taken at 24 h and 48 h after a single uniform-width scratch wound of confluent pterygium fibroblasts. Dashed lines indicate the precise location of the scratch wound. Scale bar: 50 μm. (B) Pooled quantitative data of pterygium-derived fibroblast migration. Data were summarized as the mean ± standard deviation of 3 separate experiments. ***P < 0.001 vs control.

A pure pterygium fibroblast culture was established from the explant-cultured pterygium tissue, as shown in a previous study [77]. In the present study, a scratch wound assay was performed to evaluate the effects of MMP-3 and MMP-13 inhibition on the migration of pterygium-derived fibroblasts. The scratch wound assay clearly demonstrated that an inhibitor of MMP-3 (1 μM) or MMP-13 (1 μM) retarded coverage of the uniform width of the wound area (Fig 4A). After 24 h, many cells had migrated into the wound region of the control culture, but only a few cells had migrated into the wound region in inhibitor-exposed cultures. After 48 h, control cultured cells had covered the wound area, whereas wound closure was significantly reduced in the cells exposed to an MMP-3 or MMP-13 inhibitor (Fig 4A). There were statistically significant differences between the control group and the treated groups (Fig 4B). Compared to the control culture, cell migration was 52.38% (MMP-3 inhibitor) and 51.87% (MMP-13 inhibitor) lower after 24 h, and 50.35% (MMP-3 inhibitor) and 44.72% (MMP-13 inhibitor) lower after 48 h in culture. Taken together, these data suggest that the migration of pterygium fibroblasts occurs in an MMP-dependent manner.

### Reduction of MMP-3 and MMP-13 Protein Levels Following Subconjunctival Bevacizumab Injection

**Fig 5:**
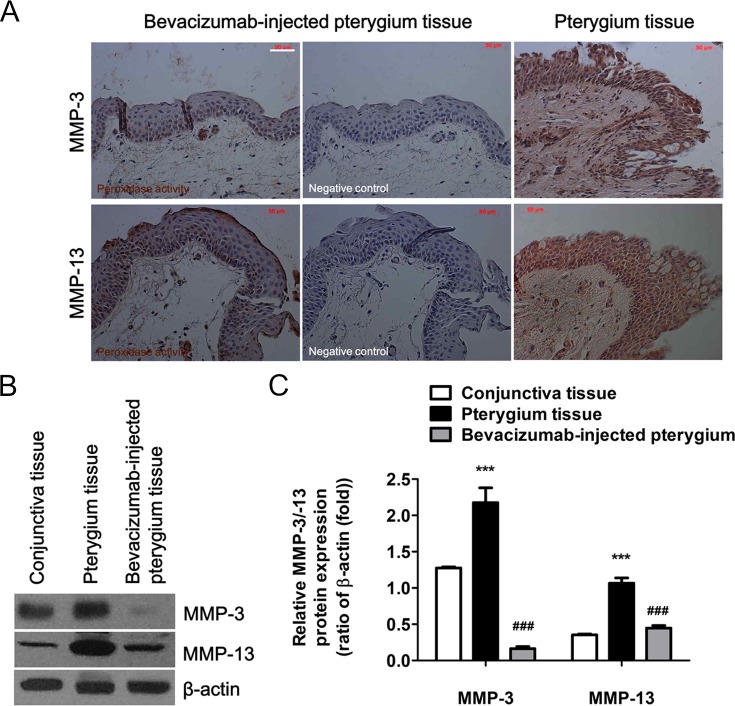
Subconjunctival administration of bevacizumab into pterygium tissues inhibited MMP-3 and MMP-13 protein expression. After injection of bevacizumab (2.5 mg/0.1 ml) for 7 days, pterygium tissues were surgically harvested. (A) Immunohistochemistry of paraffin-embedded sections (5 μm) was performed to identify MMP-3 and MMP-13 expression in bevacizumab-injected pterygium tissue (left panels) and untreated pterygium tissue (right panels). No staining was observed in negative control sections that were incubated with the secondary antibodies, but not the primary antibodies (middle panels). All tissue sections were counterstained with hematoxylin. White scale bar: 50 μm. (B) The expression of MMP-3 and MMP-13 proteins in equal amounts of cell lysate protein (30 μg) was analyzed by a western blot. β-actin was used as the loading control. (C) MMP-3 and MMP-13 band densities were quantified using ImageJ, relative to the relevant β-actin band. (B, C) Data represent the mean ± standard deviation of 3 separate experiments. ***P < 0.001 vs conjunctiva tissue; ^###^P < 0.001 vs pterygium tissue.

Changes in the expression levels of both MMP-3 and MMP-13 proteins were examined. Following subconjunctival administration of bevacizumab to pterygium patients for 7 days, immunohistochemistry was performed using excised pterygium tissues (Fig 5A). Interestingly, expression of both MMP-3 and MMP-13 proteins was significantly lower in patients treated with bevacizumab. There was no detectable expression of MMP-3 and MMP-13 proteins in the negative control sections (Fig 5A, middle panels). Next, the expression levels of MMP-3 and MMP-13 proteins were measured by western blot analysis (Fig 5B) using lysates of 3 normal conjunctiva tissues from 6 patients and of 4 pterygium tissues from 20 patients, as described in the Materials and Methods section. Higher levels of both proteins were clearly detected in pterygium tissues, as compared to those in normal conjunctiva tissues. This analysis also confirmed that the MMP-3 and MMP-13 protein expression levels decreased markedly following bevacizumab administration for 7 days (Fig 5B). These results indicate that bevacizumab mediates the down-regulation of both proteins in the pterygium tissue. There were also statistically significant differences in the expression levels of both proteins in the conjunctiva, pterygium, and bevacizumab-treated pterygium tissues (Fig 5C). Following normalization to β-actin expression, MMP-3 and MMP-13 levels increased by 1.7-fold and 3.02-fold (respectively) in pterygium tissue, as compared to their levels in conjunctiva tissue. In contrast, the intensity of both protein bands was significantly reduced in the pterygium tissues treated with bevacizumab (MMP-3, 0.13-fold; MMP-13, 1.27-fold), as compared to pterygium tissue from untreated patients (Fig 5C). These results suggest that bevacizumab exerts an important effect on cell migration that may be mediated, in part, by inhibition of MMP-3 and MMP-13 expression.

### Inhibitory Effects of Bevacizumab on MMP Expression and Pterygium Fibroblast Migration into a Scratch Wound

**Fig 6:**
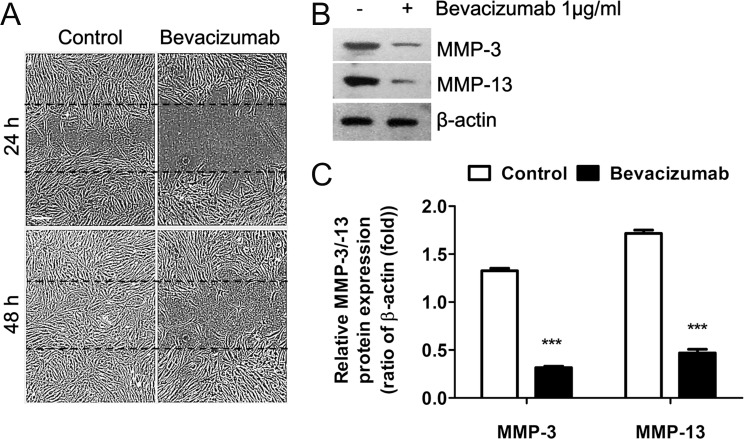
Bevacizumab-induced inhibition of scratch wound pterygium fibroblast migration and down-regulation of MMP-3 and MMP-13 expression. (A) Effects of bevacizumab (1 μg/ml) on cell migration were determined using a scratch wound assay and phase-contrast microscopy. Dashed lines indicate the precise location of the scratch wound. White scale bar: 50 μm. (B) MMP expression in these cells was determined by western blot analysis using equal amounts of cell lysate protein (30 μg), harvested at 24 h. Note that levels of both MMP-3 and MMP-13 expression were dramatically down-regulated by bevacizumab treatment. β-actin was used as the loading control. (C) Band densities were measured using ImageJ, relative to the relevant β-actin band. Data represent the mean ± standard deviation of 3 separate experiments. ***P < 0.001 vs control.

Fig 6 shows the effects of bevacizumab on cultured confluent pterygium fibroblast migration following a scratch wound, in relation to MMP-3 and MMP-13 expression. Phase-contrast microscopy (Fig 6A) of the control culture 24 h after the scratch wound (a) showed that many cells had migrated into the wound region from the wound edge; however, there were very few or no cells in the wound region of the bevacizumab-treated group (b). By 48 h, the control wound region was completely covered with fibroblasts (c), while few cells were observed in the wound region of the bevacizumab-treated group (d). Western blot analysis also showed that both MMP-3 and MMP-13 expression levels markedly decreased in the bevacizumab-treated cultures at 24 h, as compared to the high levels of both proteins observed in the control cultures (Fig 6B). The differences in MMP-3 (4.19-fold) and MMP-13 (3.66-fold) protein levels between the control and bevacizumab-treated cells were statistically significant (*P* < 0.05; Fig 6C).

### Combined Inhibitory Effect of Bevacizumab and CsA on Pterygium Fibroblast MMP Expression and Migration into a Scratch Wound

**Fig 7:**
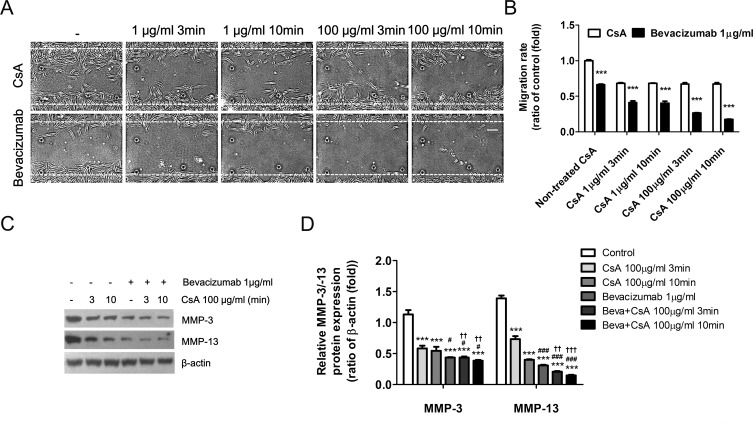
Combined Bevacizumab- and CsA-induced inhibition of scratch wound pterygium fibroblast migration and down-regulation of MMP-3 and MMP-13 expression. (A) Phase contrast microscopy of pterygium fibroblasts 24 h after scratch wounding. Cell migration in the scratched area was greatly reduced in the presence of CsA and/or bevacizumab. Dashed lines indicate the precise location of the scratch wound. White scale bar: 50 μm. (B) The migration rates were quantified in cells pretreated with CsA for 3 min or 10 min prior to incubation with bevacizumab for 24 h. Data represent the mean ± standard deviation of 3 separate experiments. ***P < 0.001 vs CsA. (C) Western blot analysis showed that expression of both MMPs was markedly reduced by combined bevacizumab and CsA treatment. (D) Band densities were analyzed using ImageJ, relative to the relevant β-actin band. Data represent the mean ± standard deviation of 3 separate experiments. ***P < 0.001 vs control; ^#^P < 0.05, ^###^ P < 0.001 vs CsA; ^††^P < 0.01, ^†††^P < 0.001 vs bevacizumab.

A previous study showed that CsA (0.05%) inhibited the proliferation of pterygium-derived fibroblasts in culture [81]. The present study therefore investigated whether CsA inhibited the migration of pterygium fibroblasts or altered MMP expression following a scratch wound. Immediately after the scratch wound was made through the middle of the confluent cultured pterygium fibroblasts, cells were pretreated with CsA (1 μg/ml or 100 μg/ml) for 3 or 10 min. These cells were then cultured in the presence or absence of bevacizumab (1 μg/ml) for 24 h (Fig 7). Interestingly, although pretreatment with CsA inhibited cell migration, as compared to control cultures, there was no difference in cell migration, regardless of treatment time or concentrations of CsA (Fig 7A). In addition, we observed that CsA pretreatment inhibited cell migration to a greater extent than bevacizumab treatment alone (Fig 7B). Furthermore, we examined the effects of CsA on MMP-3 and MMP-13 expression in pterygium fibroblasts 24 h after the scratch wound was made (Fig 7C). This western blot analysis showed that that levels of MMP-3 and MMP-13 proteins were much lower in the CsA-pretreated (100 μg/ml) groups (3 and 10 min) than in the control cultures (Fig 7C and 7D). Furthermore, cells that were CsA-pretreated and exposed to bevacizumab showed slightly lower expression levels of both MMPs. Taken together, these results suggest that CsA may inhibit the migration of pterygium fibroblasts by down-regulating both MMP-3 and MMP-13.

### Effects of Bevacizumab and CsA on Fibroblast Migration from Pterygium Explants

**Fig 8:**
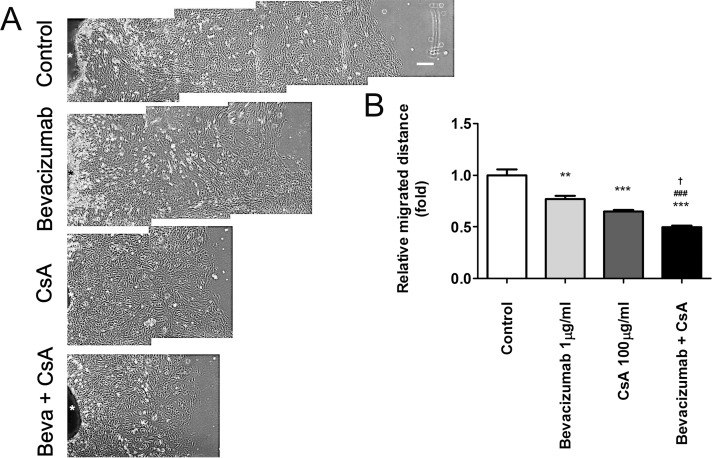
Combined bevacizumab- and CsA-induced inhibition of Cell migration from pterygium explants. (A) Cell outgrowth from explanted pterygium tissues (T) was examined for 48 h in the presence of bevacizumab (1 μg/ml) and/or following pretreatment with CsA (0.01% for 3 min). The arrow direction indicates cells migrating out from the cultured explant. White scale bar: 200 μm. (B) Migration distance quantification showing the mean ± standard deviation of 3 separate experiments. **P < 0.01, ***P < 0.001 vs control; ^###^P < 0.001 vs bevacizumab; ^†^P < 0.05 vs CsA.

Next, we examined whether bevacizumab and CsA affected fibroblast outgrowth from cultured pterygium tissue explants over 48 h (Fig 8). In the control cultures, many fibroblasts migrated out a long distance from the pterygium explant tissue. The distance migrated by the cells from the edge of the pterygium explants was markedly reduced by treatment with either bevacizumab (1 μg/ml) or pretreatment with CsA (100 μg/ml) for 3 min, as compared to control cultures. Interestingly, a greater inhibition of the migration distance was observed in the CsA-pretreated group than in the bevacizumab-treated group. Moreover, we found that the inhibitory effect of combined CsA pretreatment and bevacizumab was higher than that of the individual treatments (Fig 8A). Quantification of the migration distance demonstrated that the rate of cell migration was significantly decreased by CsA pretreatment, with or without bevacizumab (Fig 8B). Together, these data suggest that signaling events triggered by CsA or bevacizumab can play an important role in fibroblast migration.

## Discussion

It is well known that members of the MMP family of enzymes mediate cell migration by influencing ECM proteolysis and remodeling during developmental and pathogenic processes. Previous studies have demonstrated that a pterygium arises when altered limbal basal epithelial cells (pterygium cells) expressing multiple types of MMPs move onto BL and bring about the dissolution of this layer [4, 5, 26]. Activated and altered fibroblasts at the head of the pterygium primarily express MMP-1 [22]. The levels of MMPs in the pterygium and in its fibroblasts are closely related. Pterygium progression is characterized by the development of angiogenesis and the proliferation of fibrovascular tissue [82]. In the present study, we have focused on the involvement of MMP-3 and MMP-13 in pterygium pathogenesis, and identified high levels of expression of both of these MMPs in migrating pterygium fibroblasts (Fig 2). Importantly, exposure to potent inhibitors of MMP-3 or MMP-13 activity suppressed the migration of cells from cultured pterygium tissues and of cultured pterygium fibroblasts (Figs 3 and 4). Taken together, these findings suggest that MMP-3 and MMP-13 activities are both required for the migration of pterygium fibroblasts in the pathogenesis of pterygia. In addition, we demonstrated that combined treatment with bevacizumab and/or CsA down-regulated both MMP-3 and MMP-13 expression and greatly decreased pterygium fibroblast migration, confirming that these activities are necessary for this process.

### Possible Roles of MMP-3 in Pterygium Progression

Types I, II, III, and IV collagens make up a large component of the ECM material in pterygia [83, 84]. MMP-1 can degrade native fibrillar collagen types I, II, III, IX, and XI [85]. MMP-3 has a broad substrate specificity that includes casein, proteoglycans, fibronectin, elastin, and laminin, as well as collagen types III, IV, V, IX, and X [24]. Pterygium fibroblasts express mainly MMP-1 in response to cytokines and UV irradiation, as well as some MMP-3, but almost none of the other MMPs [22, 84]. Other recent studies have shown that MMP-3 levels are elevated in pterygia [5, 17]. Fibroblasts in pterygia, in areas of elastotic degradation, were mainly immunostained for both MMPs [22]. In areas of BL dissolution, as indicated by immunostaining of MMP-1 and MMP-3, pterygium fibroblasts invading the cornea are likely to play an important role in helping to dissolve BL [18, 24]. Elevated MMP-1 and -3 expression and activities were also detected in pterygium head fibroblasts [5, 24]. Following exposure to inflammatory cytokines, expression of MMP-1, MMP-2, and MMP-3 was up-regulated in cultured pterygia fibroblasts [24, 25]. MMP-3 may play a key role in ECM remodeling because it can activate other latent forms of MMPs including MMP-1, MMP-7, and MMP-9, [5, 86]. Taken together with the high levels of MMP-3 expression observed in the present study, these findings indicate that MMP-3 is a potential candidate molecule for involvement in the pathogenesis and progression of pterygium.

### Possible Roles of MMP-13 in Pterygium Progression

A previous study demonstrated the immunohistochemical localization of collagen types I, II, III, and IV in the pterygium [83]. The stromata of pterygium and normal conjunctival tissues contain collagen types I, II, and III [83]. The expression of MMP-13 appears to be relatively restricted and confined to a few normal tissues [87]. MMP-13 can readily cleave the triple helical domain of native fibrillar collagens I, II, and III, as well as other ECM molecules [3]. A previous study reported that the staining intensity of MMP-13 was greater in pterygium epithelial cells than in pterygium fibroblasts [26]. On the other hand, consistent with our immunohistochemical data shown in Fig 1, another study identified strong MMP-13 expression over the entire pterygium stroma and epithelium [3]. The expression of MMP-13 was also detected at the leading edge of pterygia [26]. Variation in the expression of MMPs may explain the differences in the growth of pterygia seen clinically [17]. A characteristic feature of pterygia is the loss of BL, presumably due to over-expression of MMPs [26]. MMP-13 was stained intensely in the basal and columnar epithelium, particularly in the regions of fragmented BL, adjacent to pterygium fibroblasts [26]. Therefore, we suggest that MMP-13 expressed by pterygium tissues may induce collagen II and III remodeling in pterygium stroma. Furthermore, our results suggest that pterygium recurrence *in vivo* results from uncontrolled degradation of the ECM caused by an excess of MMP-13 in pterygium tissues.

### Inhibition of Pterygium Fibroblast Migration by Bevacizumab-induced Down-regulation of MMP-3 and MMP-13

MMP-3 has been clearly implicated in angiogenesis because it generates angiostatin, whereas its inhibitor inhibits angiogenic responses [32–34]. In addition, MMP-3 has been shown to induce vascular endothelial cell growth or migration [5, 88]. MMP-13 also mediates collagenolysis-dependent angiogenesis *in vivo* [39]. MMP-13 acts as a stromal mediator in controlling persistent angiogenesis in skin carcinoma [44]. MMP-13 also promotes the secretion of VEGF-A from fibroblasts and endothelial cells, and it is suggested that MMP-13 may directly and indirectly promote tumor invasion and angiogenesis both *in vitro* and *in vivo* [38]. In addition, MMP-13 has been implicated in corneal vascularization [43]. On the other hand, diminished expression of MMP-13 in hypertrophic chondrocytes inhibits growth-plate angiogenesis [42]. Lack of MMP-13 reduces the vascular density of wound granulation tissue in mouse skin [41]. Importantly, the present study found that treatment with MMP-3 or MMP-13 inhibitors reduced the migration of fibroblasts from cultured pterygium tissues and into a scrape wound (Figs 3 and 4).

VEGF is known to have a role in angiogenesis and is markedly elevated in pterygia, as compared with normal conjunctival samples [28–31]. In addition, VEGF is secreted at high levels by human pterygium fibroblasts [89]. Bevacizumab (Avastin) is an angiogenesis inhibitor that slows the growth of new blood vessels. Avastin treatment decreased human pterygium fibroblast migration and invasion [89]. Many previous studies have shown that subconjunctival bevacizumab injections are useful in the management of patients with recurrent pterygium, without significant local or systemic adverse effects. Bevacizumab has been shown to decrease both angiogenesis and pterygium recurrence [57, 90–94]. The present *in vivo* and *in vitro* bevacizumab results (Figs 5, 6, 7 and 8) indicate that there was a strong correlation between the inhibition of MMP-3 and MMP-13 expression by bevacizumab and the inhibition of pterygium fibroblast migration. Interestingly, a previous study showed that increased levels of MMPs promoted dissolution of the BL and promoted both angiogenesis and pterygium invasion of the cornea [27]. To date, there are no MMP studies that relate to pterygium recurrence. Although the detailed mechanisms involved in bevacizumab-mediated inhibition of MMP-3 and MMP-13 expression and pterygium fibroblast migration are currently unknown, the present study suggests that bevacizumab could make an important contribution to the management of pterygia. Further study will be necessary in order to elucidate the mechanism underlying this effect.

### Combined Effects of CsA and Bevacizumab on MMP-3, MMP-13, and Pterygium Fibroblast Migration

The first-line treatment for pterygium is surgical excision, but recurrence is the most common complication [63]. CsA, an anti-inflammatory agent, has been administered topically to treat a variety of inflammatory disorders of the ocular surface [60]. Postoperative application of topical 0.05% CsA can provide effective prevention of recurrence after primary pterygium surgery [64, 65, 95]. To the best of our knowledge, no previous studies have examined the relationship between MMP-3 and MMP-13 expression by pterygium explants and cultured pterygium-derived fibroblasts, and treatment using bevacizumab and/or CsA. Interestingly, similar to the bevacizumab effects, CsA produced a significant reduction in MMP-3 and MMP-13 expression and in pterygium fibroblast migration (Figs 7 and 8). Importantly, combined CsA and bevacizumab produced a larger inhibition of MMP-3 and MMP-13 expression and of pterygium fibroblast migration, as compared to treatment with bevacizumab or CsA alone (Figs 7 and 8). Therefore, we suggest that bevacizumab and CsA produced synergistic down-regulation of MMP expression and pterygium cell migration. UV-inducible cytokines generated by the pterygium epithelium, such as interleukins and tumor necrosis factor (TNF)-α, may contribute to the initiation of neovascularization and chronic inflammation during pterygium formation [84, 96]. Interleukin-6 has been shown to increase angiogenesis via VEGF induction [97]. Although previous studies suggest that CsA prevents the synthesis and secretion of interleukins [98, 99] and TNF-α [100], and blocks VEGF-induced angiogenesis [99, 101, 102], the mechanisms involved in CsA-mediated inhibition of MMP expression and pterygium fibroblast migration have not been completely elucidated. However, the present data suggest that increased expression of MMP-3 and MMP-13 by pterygium fibroblasts may be involved in the pathogenesis of pterygium, and combination treatment with bevacizumab and CsA therefore represents a potential therapy for pterygium recurrence.

In summary, our study demonstrated for the first time that the combined actions of CsA and bevacizumab produced stronger inhibitory effects on MMP expression and *in vitro* pterygium fibroblast migration than either intervention alone. Therefore, the combined application of CsA and bevacizumab could provide a useful alternative approach to the clinical management of pterygium recurrence. Further studies will be necessary to understand the precise molecular mechanisms underlying these effects.

## Supporting Information

S1 Fig Inhibition of cell migration by treatment of VEGF inhibitors (bevacizumab, aflibercept and ranibizumab) in scratch wound pterygium fibroblasts. Effects of bevacizumab (1 μg/ml), aflibercept (a recombinant fusion protein which binds all VEGF-A isoforms, 1 μg/ml), and ranibizumab (a monoclonal antibody fragment (Fab) created from the same parent mouse antibody as bevacizumab, 1 μg/ml) on cell migration were determined by using a scratch-wound healing migration assay. (A) By phase-contrast microscopy, compared to control cultured cells, cell migration was markedly blocked in pterygium fibroblast cells by treatment with bevacizumab, aflibercept, and ranibizumab at 24 h and 48 h. Dashed lines indicate the precise location of the scratch wound. (B) The quantification of migration length and migrated cell numbers. Note that compare to control culture, cell migration was significantly inhibited by all treatment groups. As expected, migrated cell numbers were increased in control cultures for 24 h and 48 h, compared to all treatment groups. Data were summarized as mean ± SD from 3 separated experiments. ***P < 0.001 vs control.
(TIF)
