# Supplementary figures and images for: Inhibition of Pterygium Fibroblast Migration and Outgrowth by Bevacizumab and Cyclosporine A Involves Down-Regulation of Matrix Metalloproteinases-3 and -13
Source: PLoS One. 2017 Jan 9;12(1):e0169675. doi: 10.1371/journal.pone.0169675 (PMC5221804; doi:10.1371/journal.pone.0169675)

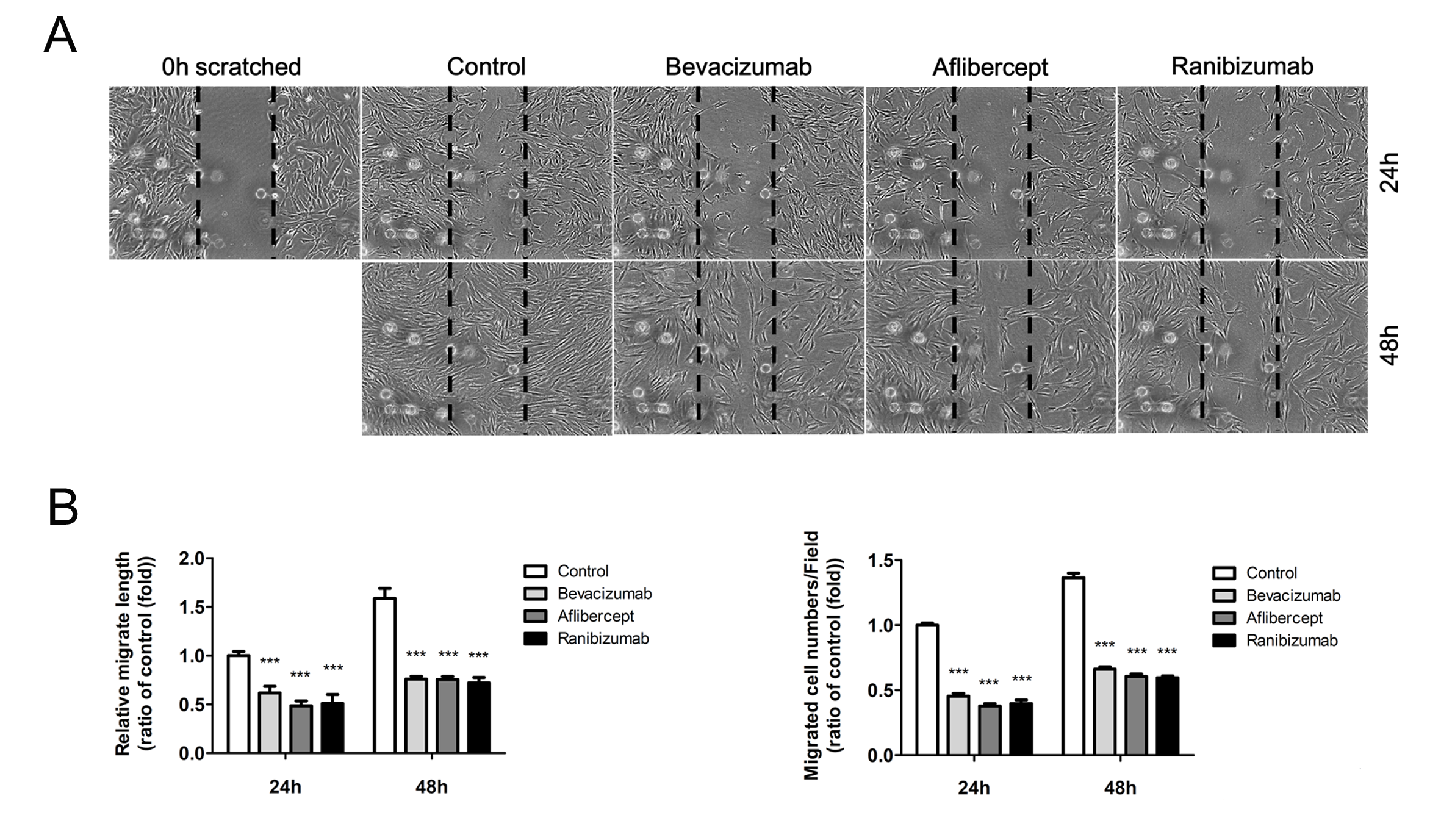

Supplement: S1 Fig — Effects of bevacizumab (1 μg/ml), aflibercept (a recombinant fusion protein which binds all VEGF-A isoforms, 1 μg/ml), and ranibizumab (a monoclonal antibody fragment (Fab) created from the same parent mouse antibody as bevacizumab, 1 μg/ml) on cell migration were determined by using a scratch-wound healing migration assay. (A) By phase-contrast microscopy, compared to control cultured cells, cell migration was markedly blocked in pterygium fibroblast cells by treatment with bevacizumab, aflibercept, and ranibizumab at 24 h and 48 h. Dashed lines indicate the precise location of the scratch wound. (B) The quantification of migration length and migrated cell numbers. Note that compare to control culture, cell migration was significantly inhibited by all treatment groups. As expected, migrated cell numbers were increased in control cultures for 24 h and 48 h, compared to all treatment groups. Data were summarized as mean ± SD from 3 separated experiments. ***P < 0.001 vs control. (TIF) [file pone.0169675.s001.tif]
